# Clinicopathological Significance of Epithelial–Mesenchymal Transition in Oral Squamous Cell Carcinoma

**DOI:** 10.3390/ijms27115002

**Published:** 2026-06-01

**Authors:** Paloma Lequerica-Fernández, Carmen Vallina-Fernández-Kelly, Juan Pablo Rodrigo, Rosa María López-Pintor, Héctor E. Torres-Rivas, Tania Rodríguez-Santamarta, Verónica Blanco-Lorenzo, Saúl Álvarez Teijeiro, Juana M. García-Pedrero, Juan Carlos de Vicente

**Affiliations:** 1 Instituto de Investigación Sanitaria del Principado de Asturias (ISPA), Instituto Universitario de Oncología del Principado de Asturias (IUOPA), Universidad de Oviedo, C/Carretera de Rubín, s/n, 33011 Oviedo, Asturias, Spain; palomalequerica@gmail.com (P.L.-F.); jprodrigo@uniovi.es (J.P.R.); taniasantamarta@gmail.com (T.R.-S.); veronica.blanco@sespa.es (V.B.-L.); saul.teijeiro@gmail.com (S.Á.T.); 2 Department of Biochemistry, Hospital Universitario Central de Asturias (HUCA), C/Carretera de Rubín, s/n, 33011 Oviedo, Asturias, Spain; 3 Department of Oral and Maxillofacial Surgery, Hospital Universitario Central de Asturias (HUCA), C/Carretera de Rubín, s/n, 33011 Oviedo, Asturias, Spain; carmenvallinafkelly@hotmail.com; 4 Department of Otolaryngology, Hospital Universitario Central de Asturias (HUCA), C/Carretera de Rubín, s/n, 33011 Oviedo, Asturias, Spain; 5 Department of Surgery, Universidad de Oviedo, 33011 Oviedo, Asturias, Spain; 6 Centro de Investigación Biomédica en Red de Cáncer (CIBERONC), Instituto de Salud Carlos III, Av. Monforte de Lemos, 3-5, 28029 Madrid, Spain; 7 ORALMED Research Group, Department of Dental Clinical Specialties, School of Dentistry, Complutense University of Madrid, 28040 Madrid, Spain; rmlopezp@odon.ucm.es; 8 Department of Pathology, Hospital Universitario Central de Asturias (HUCA), C/Carretera de Rubín, s/n, 33011 Oviedo, Asturias, Spain; ress_444@yahoo.com

**Keywords:** epithelial–mesenchymal transition, cadherin switching, vimentin, snail, slug

## Abstract

Epithelial–mesenchymal transition (EMT) contributes to oral squamous cell carcinoma (OSCC) progression, although the clinical relevance of EMT-related markers remains controversial. This study evaluated the expression of EMT markers and transcription factors and their associations with clinicopathological features and survival in OSCC. Immunohistochemical expression of E-cadherin, N-cadherin, vimentin, and EMT-associated transcription factors (Snail1, Slug, Twist, ZEB1, ZEB2, and E47) was assessed in OSCC samples. Associations with clinicopathological variables were analyzed using χ^2^ or Fisher’s exact test, while survival outcomes were evaluated using Kaplan–Meier and Cox regression analyses. E-cadherin, vimentin, and N-cadherin were expressed in 54.5%, 39.6%, and 2.5% of cases, respectively. Loss of E-cadherin and vimentin expression was associated with adverse clinicopathological features, including advanced stage, lymph node metastasis, and poor differentiation. Vimentin expression was significantly associated with reduced disease-specific and overall survival in univariate analysis. Based on combined E-cadherin and vimentin expression, tumors were classified as non-EMT, partial EMT, or complete EMT. Complete EMT was associated with significantly poorer survival, whereas partial EMT showed no prognostic impact. Vimentin independently predicted disease-specific survival, while EMT-related alterations overall appeared strongly influenced by established clinicopathological factors, particularly tumor stage.

## 1. Introduction

Oral squamous cell carcinoma (OSCC), the most common histopathological form of oral cancer, is among the most prevalent cancers worldwide [1]. It has a 5-year survival rate ranging from 15% to 60%, depending on the stage of the disease, largely due to its tendency for local invasion and metastasis, the most lethal consequence of tumor progression [2]. Epithelial–mesenchymal transition (EMT) is a biological process in which immotile epithelial cells undergo morphological and phenotypic changes to acquire a mesenchymal, spindle-shaped, and motile phenotype [3]. EMT plays a crucial role in embryonic morphogenesis; although it is largely silenced in adult tissues, it can be reactivated under pathological conditions, including carcinomas. It has been hypothesized that EMT confers an invasive phenotype on cancer cells, enabling them to invade surrounding tissues and eventually disseminate through blood or lymphatic vessels to form metastases [4]. However, most evidence supporting EMT is derived from experimental models [5,6,7,8], and its role in human cancer remains controversial [9,10,11,12]. EMT and has been associated with poor survival in several cancers, including head and neck cancer [13]. Nevertheless, its prognostic significance in OSCC remains controversial [14,15,16].

EMT-associated phenotypic alterations are characterized by the loss of epithelial cell junction proteins and the acquisition of mesenchymal features, including the expression of mesenchymal markers such as vimentin. A hallmark of this process is the so-called “cadherin switching”, typically involving the loss of E-cadherin and gain of N-cadherin expression. Rather than a strictly binary epithelial-to-mesenchymal transition, EMT is currently recognized as a dynamic and reversible spectrum. Tumor cells may acquire intermediate or hybrid epithelial–mesenchymal phenotypes, commonly referred to as partial EMT (pEMT), which retain both epithelial and mesenchymal traits and have been associated with tumorigenesis, invasion, metastasis, immune modulation, and therapeutic resistance [14,17]. Based on the combined expression of E-cadherin and vimentin, Wangmo et al. [15] classified EMT into three categories: (1) no EMT (E-cadherin-positive and vimentin-negative), (2) complete EMT (E-cadherin-negative and vimentin-positive), and (3) partial EMT (co-expression or absence of both markers).

The completion of EMT requires activation of a genetic program that not only represses E-cadherin expression but also induces mesenchymal gene expression. A recent meta-analysis has identified several key genes involved in EMT induction [18], and cigarette smoking has been shown to promote EMT through upregulation of EMT-related genes [19]. Zinc finger proteins Snail1 (Snail) and Snail2 (Slug), Twist, E47, and zinc finger E-box-binding homeobox 1 and 2 (ZEB1 and ZEB2) are transcription factors capable of inducing EMT (EMT-TFs) [20,21]. However, their roles in OSCC progression remain unclear. These factors suppress E-cadherin expression, primarily through promoter methylation or through the upregulation of EMT-related transcriptional regulators [16]. In addition, Twist upregulates N-cadherin expression [20], while Snail promotes the expression of mesenchymal genes such as vimentin, thereby enhancing tumor invasiveness. The basic helix–loop–helix transcription factor E47 is also an EMT inducer, capable of regulating the transcription of fibronectin and cytokines such as tumor necrosis factor Alpha [22].

Although EMT-related markers have been individually investigated in OSCC, findings remain heterogeneous and often inconsistent, particularly regarding their clinicopathological and prognostic significance. Moreover, most studies have focused on a limited number of markers rather than simultaneously evaluating a broad EMT-related panel, including both structural proteins and EMT-associated transcription factors. Therefore, the present study aimed to comprehensively assess the expression of multiple EMT-related proteins and their relationship with clinicopathological characteristics and patient outcomes in OSCC.

## 2. Results

### 2.1. Immunohistochemical Analysis of EMT Markers in OSCC

**Figure 1:**
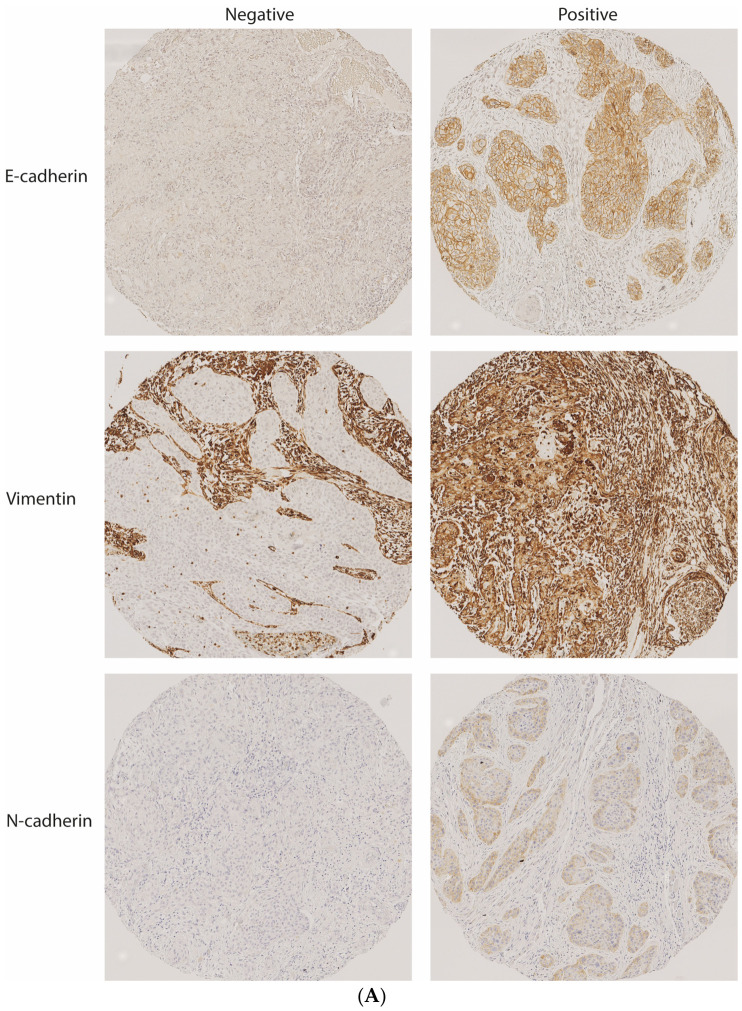

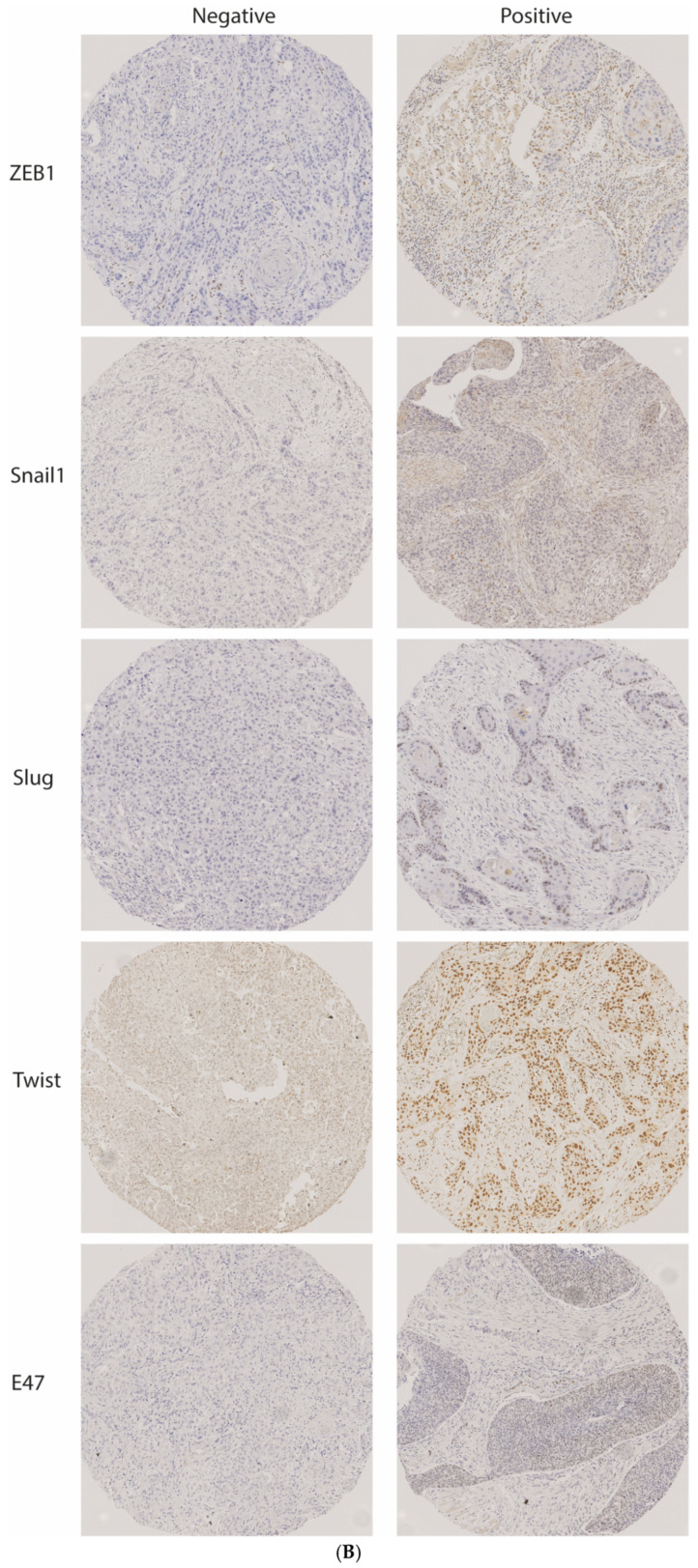
(**A**). Representative immunohistochemical staining patterns of EMT markers (E-cadherin, vimentin, and N-cadherin) in OSCC. (**B**). Representative immunohistochemical staining patterns of EMT-associated transcription factors (ZEB1, Snail1, Slug, Twist, and E47) in OSCC.

E-cadherin expression was detected in 89 (54.5%) OSCC cases, whereas vimentin and N-cadherin expression were observed in 65 (39.6%) and 4 (2.5%) cases, respectively. Five cases were not evaluable for N-cadherin, and two cases were not evaluable for E-cadherin and vimentin. Regarding EMT-associated transcription factors, Twist immunoreactivity was predominantly localized to tumor cell nuclei, Snail1 showed both nuclear and cytoplasmic staining, whereas Slug expression was mainly cytoplasmic. Snail1 expression was detected in 98 (59.4%) tumors, Slug in 136 (82.4%), ZEB1 in 5 (3.0%), Twist in 155 (94.5%), and E47 in 7 (4.2%). In contrast, ZEB2 expression was not detected in any tumor sample. One case was not evaluable for EMT transcription factor analysis, except for Twist, for which two cases were non-evaluable. Representative immunohistochemical staining patterns of EMT markers (E-cadherin, N-cadherin, and vimentin) and EMT-associated transcription factors (Snail1, Slug, ZEB1, Twist, and E47) are shown in Figure 1A,B.

### 2.2. Associations Among EMT Markers and Transcription Factors

A significant association was observed between loss of E-cadherin expression and vimentin positivity (*p* = 0.004). E-cadherin expression was also significantly associated with Snail1 (*p* = 0.04) and Slug expression (*p* < 0.0001). Significant correlations were additionally identified between Snail1 and Slug expression (*p* < 0.0001), as well as between Slug and vimentin expression (*p* = 0.04). Furthermore, Slug expression was significantly associated with Twist expression (*p* < 0.0001), whereas ZEB1 expression was significantly associated with E47 expression (*p* < 0.0001).

### 2.3. Associations with Clinicopathological Variables and Prognosis

Table 1 summarizes the associations between EMT markers and clinicopathological characteristics. Loss of E-cadherin expression was significantly associated with advanced clinical stage (χ^2^ test, *p* = 0.03), whereas N-cadherin expression was associated with moderately or poorly differentiated tumors (Fisher’s exact test, *p* = 0.01). Vimentin expression was significantly associated with cervical lymph node metastasis (*p* = 0.002), advanced clinical stage (*p* = 0.009), moderate or poor tumor differentiation (*p* = 0.001), tumor recurrence (*p* = 0.001), and disease-specific mortality (*p* = 0.001).

Regarding EMT-associated transcription factors, Snail1 expression was significantly associated with male sex (*p* = 0.02) and tobacco consumption (*p* = 0.01). Slug expression was associated with tobacco use (*p* = 0.04) and alcohol consumption (*p* = 0.02), whereas an inverse association was observed with tumor classification (*p* = 0.001) and clinical stage (*p* = 0.002). No significant associations were identified between ZEB1, Twist, or E47 expression and the clinicopathological variables analyzed (Table 2).

### 2.4. Survival Analysis

At the last follow-up (range: 1–221 months), 67 patients (40.4%) had died from OSCC-related disease. The mean and median follow-up times were 62.6 months (SD: 53.6) and 59.0 months, respectively. The 5- and 10-year disease-specific survival (DSS) rates were 61% and 53%, respectively, with a mean DSS time of 132.7 months (95% CI: 116.5–149.0). Tumor classification (T1–2 vs. T3–4), lymph node status (N0 vs. N+), and clinical stage (I–II vs. III–IV) were all significantly associated with DSS (*p* < 0.0005), with hazard ratios of 3.9, 3.8, and 4.3, respectively.

**Figure 2:**
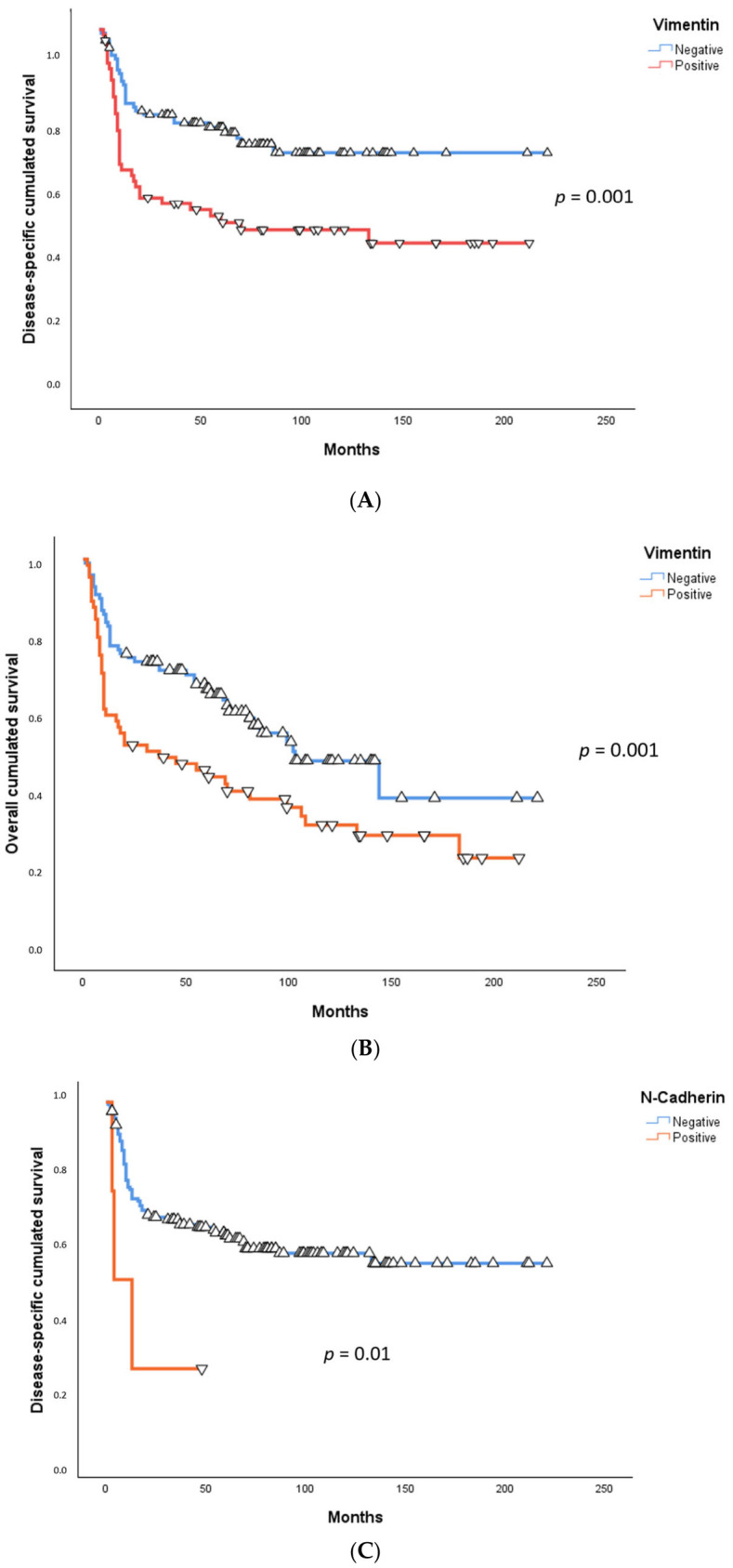

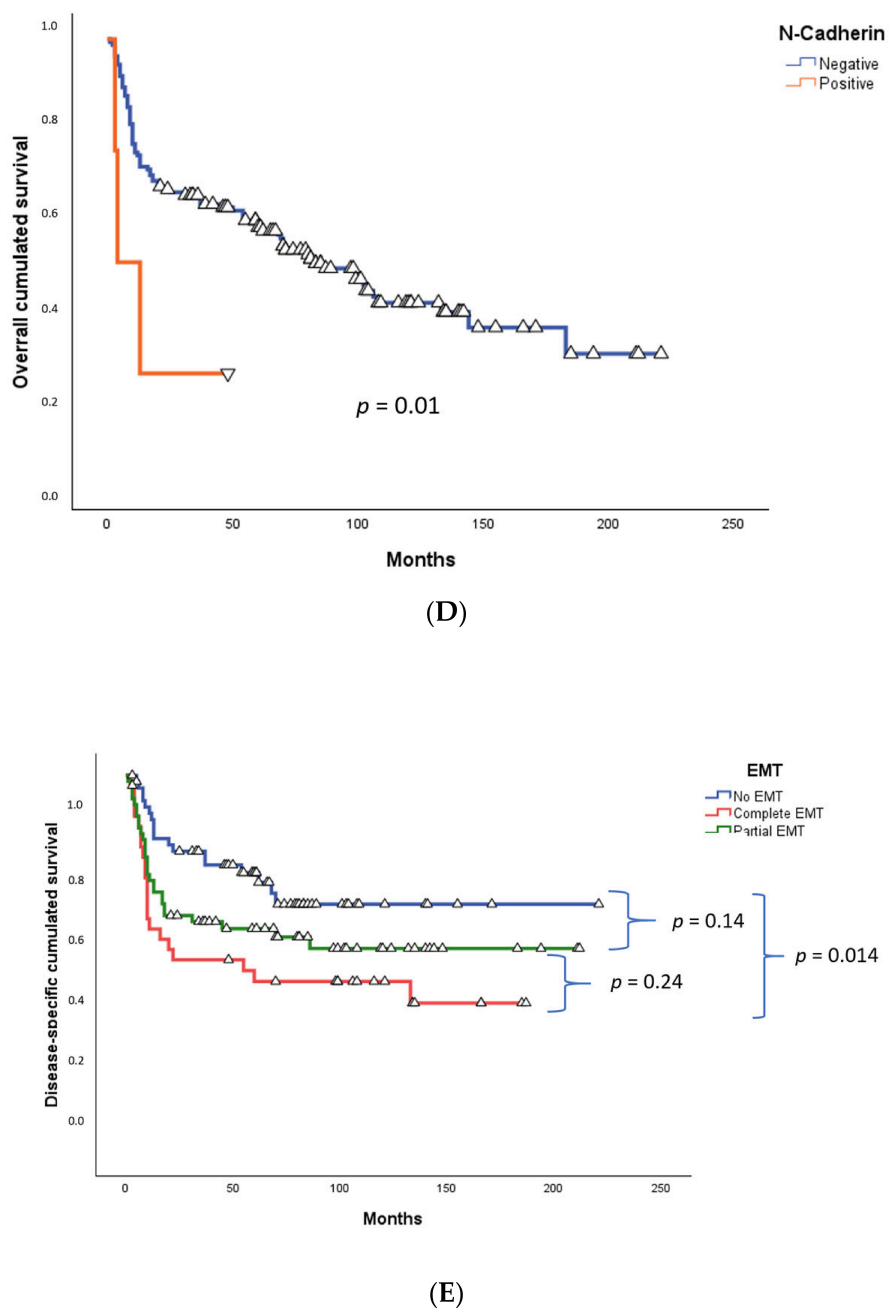
(**A**). Disease-specific cumulated survival according to vimentin expression in 165 OSCC patients. Log-rank test results are shown. (**B**). Overall cumulated survival according to vimentin expression in 165 OSCC patients. Log-rank test results are shown. (**C**). Disease-specific cumulated survival according to N-cadherin expression in 165 OSCC patients. Log-rank test results are shown. (**D**). Overall cumulated survival according to N-cadherin expression in 165 OSCC patients. Log-rank test results are shown. (**E**). Disease-specific cumulated survival according to EMT classification in 165 OSCC patients. Log-rank test results are shown.

Kaplan–Meier survival analysis demonstrated that patients with positive vimentin expression had significantly reduced DSS (*p* = 0.001; HR = 2.18; 95% CI: 1.34–3.55) (Figure 2A) and overall survival (OS) (*p* = 0.001; HR = 1.77; 95% CI: 1.16–2.71) (Figure 2B).

Consistent with vimentin expression, positive N-cadherin expression was associated with significantly shorter DSS (*p* = 0.01; HR = 3.63; 95% CI: 1.13–11.65) (Figure 2C) and OS (*p* = 0.01; HR = 3.29; 95% CI: 1.03–10.54) (Figure 2D).

Neither E-cadherin expression nor any of the EMT-associated transcription factors analyzed showed significant associations with survival outcomes.

Based on EMT classification, 63 tumors (38%) were categorized as non-EMT, 64 (39%) as partial EMT, and 38 (23%) as complete EMT. EMT status was significantly associated with advanced clinical stage (*p* = 0.001), poorer tumor differentiation (*p* = 0.03), and disease-specific mortality (*p* = 0.04).

Kaplan–Meier survival and Cox regression analyses demonstrated that complete EMT was significantly associated with reduced disease-specific survival compared with non-EMT tumors (mean survival: 90.2 vs. 153.9 months; *p* = 0.014; HR = 2.18; 95% CI: 1.17–4.06) (Figure 2E). No significant differences in DSS were observed between partial EMT and non-EMT tumors (*p* = 0.14) or between partial and complete EMT tumors (*p* = 0.24).

In a multivariate model including vimentin and N-cadherin, vimentin remained an independent predictor of DSS (*p* = 0.001; HR = 2.21; 95% CI: 1.36–3.60) and OS (*p* = 0.005; HR = 1.83; 95% CI: 1.20–2.80).

In a second model adjusted for clinical stage (*p* < 0.001; HR = 3.86; 95% CI: 2.22–6.72), vimentin remained independently associated with DSS (*p* = 0.04; HR = 1.66; 95% CI: 1.01–2.73) but not with OS (*p* = 0.09), which was mainly driven by clinical stage (*p* < 0.001; HR = 2.66; 95% CI: 1.70–4.16).

## 3. Discussion

EMT has emerged as a relevant biological framework to explain the heterogeneity of tumor aggressiveness in oral squamous cell carcinoma (OSCC) beyond conventional clinicopathological parameters. Although prognosis still primarily relies on tumor stage, nodal status, and histological grade, these variables incompletely capture the biological behavior of individual tumors. Therefore, the identification of reliable biomarkers reflecting tumor invasiveness and progression remains of considerable clinical interest [23].

E-cadherin, a key mediator of epithelial cell–cell adhesion, is frequently downregulated during EMT, whereas N-cadherin has been associated with mesenchymal transition, invasiveness, and tumor progression [24,25]. In the present study, E-cadherin expression was detected in 54.5% of tumors, while N-cadherin expression was rare (2.5%). Loss of E-cadherin was associated with advanced clinical stage but not with survival outcomes, consistent with the heterogeneous findings reported in previous studies, which range from favorable prognostic associations to lack of independent prognostic significance in multivariate analyses [23,24,26,27,28,29,30]. The low prevalence of N-cadherin expression in our cohort suggests that classical cadherin switching may not represent a predominant mechanism of OSCC progression. This observation aligns with previous reports indicating that loss of epithelial adhesion, rather than acquisition of N-cadherin expression, may constitute the most relevant EMT-related alteration in OSCC [28,31,32]. However, the very low frequency of N-cadherin positivity limits the robustness of statistical analyses involving this marker and warrants cautious interpretation of its prognostic implications.

Among the EMT-related markers analyzed, vimentin demonstrated the strongest association with aggressive tumor behavior. Its expression correlated with lymph node metastasis, advanced clinical stage, poor differentiation, recurrence, and reduced survival. Moreover, vimentin remained an independent prognostic factor in multivariate models including EMT markers, although this association was attenuated after adjustment for clinical stage. Beyond serving as a mesenchymal marker, vimentin may contribute to cytoskeletal remodeling, cellular motility, and invasive capacity, potentially explaining its association with nodal dissemination and unfavorable outcomes. Collectively, these findings reinforce the biological and clinical relevance of vimentin in OSCC progression and are consistent with previous reports linking mesenchymal differentiation to aggressive tumor phenotypes [23].

Based on combined E-cadherin and vimentin expression, tumors were classified into non-EMT, partial EMT, and complete EMT phenotypes. Complete EMT was associated with poorer survival outcomes, whereas partial EMT showed no significant prognostic impact. These findings support the concept that EMT in OSCC may occur along a continuum of epithelial–mesenchymal plasticity (EMP) rather than as a binary process. Although partial EMT states have been proposed as highly metastatic phenotypes in experimental models [25], our results suggest that more complete mesenchymal reprogramming may be associated with adverse clinical outcomes in OSCC. Furthermore, the heterogeneous expression patterns observed across EMT-related markers support the view that EMT is dynamic and context-dependent, reflecting coexistence of epithelial and mesenchymal traits both within and between tumors [33,34,35].

EMT is orchestrated by a complex network of transcription factors, including Snail, Slug, Twist, ZEB1, ZEB2, and E47, which repress epithelial differentiation programs and promote mesenchymal features [36,37,38]. In our cohort, Snail1 and Slug were the most frequently expressed transcription factors and showed associations with smoking and selected clinicopathological variables. By contrast, Twist exhibited nearly universal expression, while ZEB2 was entirely absent, limiting their discriminatory and prognostic utility. The restricted variability observed for some transcription factors may reflect intrinsic biological characteristics of OSCC, but methodological factors related to immunohistochemical sensitivity and threshold selection should also be considered. Importantly, smoking was associated with increased Snail1 and Slug expression, supporting experimental evidence suggesting that tobacco-related oxidative stress and inflammatory signaling may promote EMT activation and tumor invasiveness [39,40,41].

From a clinical perspective, although several EMT-related markers were associated with survival in univariate analyses, their prognostic value was not independent of established clinicopathological variables, particularly tumor stage. This finding suggests that EMT-related alterations may primarily reflect underlying tumor aggressiveness rather than provide fully independent prognostic information beyond conventional staging systems.

Several limitations should be acknowledged. First, the retrospective design may introduce selection bias. Second, tissue microarray (TMA) analysis provides limited spatial representation of tumor heterogeneity and may inadequately capture EMT dynamics at the invasive front, despite the inclusion of multiple tumor cores to reduce sampling bias. Third, the binary scoring system used for immunohistochemical assessment may oversimplify the continuous and dynamic nature of EMT-related marker expression. In addition, the low prevalence of some markers, particularly N-cadherin, reduced statistical power for subgroup analyses. Finally, the absence of transcriptomic or functional validation limits mechanistic interpretation, and future studies integrating molecular approaches are warranted to better characterize EMT dynamics in OSCC [42].

Overall, our findings indicate that EMT-related alterations, particularly vimentin expression and complete EMT phenotype, are associated with increased aggressiveness in OSCC. However, their prognostic utility appears to be strongly influenced by established clinicopathological parameters, especially clinical stage, which currently remains the most robust predictor of patient outcome.

## 4. Materials and Methods

### 4.1. Patients and Tissue Specimens

A sample size of 136 patients was estimated to evaluate the association between immunohistochemical biomarkers, clinicopathological variables, and survival outcomes, assuming a group allocation ratio of 1.3, a significance level (α) of 0.05, and a statistical power (1 − β) of 0.80. To compensate for potential missing data and technical failures, the sample size was increased by 20%. Accordingly, surgical tissue specimens from 165 patients with histologically confirmed oral squamous cell carcinoma (OSCC) who underwent curative surgery at the Hospital Universitario Central de Asturias between 1 March 2000 and 31 December 2010 were retrospectively collected according to institutional review board guidelines. Clinicopathological data were obtained from medical records.

All procedures were conducted in accordance with the Declaration of Helsinki and approved by the Institutional Ethics Committee of the Hospital Universitario Central de Asturias and the Regional Ethics Committee of the Principado de Asturias (approval no. 136/19; approval date: 14 May 2019; project PI19/01255). Due to the retrospective nature of the study, the requirement for written informed consent was waived. The inclusion criteria were: (i) histopathological diagnosis of OSCC and (ii) radical resection of the primary tumor with simultaneous neck lymph node dissection. Tumors were staged according to the 8th edition of the AJCC Cancer Staging Manual.

Tissue specimens were provided by the Principado de Asturias BioBank (PT17/0015/0023), integrated within the Spanish National Biobanks Network. Representative tumor areas from archival formalin-fixed paraffin-embedded (FFPE) tissue blocks were selected for tissue microarray (TMA) construction.

### 4.2. Immunohistochemistry (IHC)

Three cores measuring 1 mm in diameter were obtained from each tumor specimen and arrayed into recipient TMA blocks. Sections (3 μm thick) were mounted on Flex IHC microscope slides (DakoCytomation, Glostrup, Denmark). After deparaffinization in xylene and rehydration through graded alcohols, antigen retrieval was performed using EnVision Flex Target Retrieval Solution (pH 9) for 20 min at 95 °C in a PT Link system (Dako). Immunohistochemical staining was performed at room temperature using an automated staining platform (Dako Autostainer Plus, Dako) with the following primary antibodies: mouse monoclonal anti-E-cadherin (Clone 36, BD Biosciences, Franklin Lakes, NJ, USA, #610181; 1:4000), mouse monoclonal anti-N-cadherin (Novus Biologicals, Centennial, CO, USA; #13A9 NBP1-48309; 1:100), mouse monoclonal anti-vimentin (Clone RV202, Abcam, Cambridge, UK, #ab8978; 1:200), mouse monoclonal anti-Snail1 (L70G2, Cell Signaling Technology, Danvers, MA, USA, #3895; 1:200), rabbit monoclonal anti-Snail2/Slug (C19G7, Cell Signaling Technology, Danvers, MA, USA, #9585; 1:200), mouse monoclonal anti-Twist (10E4E6, Abcam, Cambridge, UK, #ab175430; 1:500), mouse monoclonal anti-ZEB1 (Clone CL0151, Novus Biologicals, Centennial, CO, USA, #NBP2-52866; 1:200), rabbit monoclonal anti-ZEB2 (Novus Biologicals, Centennial, CO, USA, #NBP1-82991; 1:200), and rabbit polyclonal anti-E47 (TCF3; Invitrogen, Thermo Fisher, Waltham, MA, USA, #PA5-84553; 1:200). Detection was carried out using the Dako EnVision Flex+, Glostrup, Denmark, visualization system with diaminobenzidine as chromogen. Negative controls were prepared by omitting the primary antibody. Sections were counterstained with hematoxylin. Positive external controls were included for all antibodies to confirm assay performance. Slides were scanned using a NanoZoomer-SQ Digital Slide Scanner C13140-01 (Hamamatsu) and analyzed with NDP.scan and NDP.View software (version 1.0.9).

Immunohistochemical evaluation was independently performed by three observers (HET-R, VB-L, and JPR) blinded to the clinicopathological data. Discrepant cases were reviewed jointly until consensus was reached. Since no universally accepted immunohistochemical cutoff values exist for epithelial–mesenchymal transition (EMT) markers, the scoring criteria were established according to previously published studies. Membranous staining was considered positive for E-cadherin. E-cadherin expression was initially scored as follows: 0, no stained cells; 1, <10% positive cells; 2, 10–75% positive cells; and 3, >75% positive cells [43]. For statistical analyses, E-cadherin expression was dichotomized as negative (<10% positive cells) or positive (≥10% positive cells). N-cadherin expression was considered positive when cytoplasmic and/or membranous staining was observed in >10% of tumor epithelial cells [44]. Vimentin expression was evaluated according to the percentage of positively stained tumor cells as follows: 0, no positive cells; 1, ≤10%; 2, 10–50%; 3, 50–80%; and 4, ≥80% positive cells [45]. For statistical purposes, vimentin expression was classified as positive when >10% of tumor cells showed cytoplasmic staining. The immunoreactivity of EMT-associated transcription factors (Snail1, Slug, ZEB1, ZEB2, Twist, and E47) was semiquantitatively classified into five categories: negative (−), focal/very weak staining, weak (+), moderate (++), and strong (+++) staining [20]. For analytical purposes, any unequivocal nuclear staining was considered positive. Accordingly, all biomarkers were ultimately dichotomized as negative or positive for statistical analysis.

### 4.3. Statistical Analysis

Statistical analyses were performed using IBM SPSS Statistics for Windows (version 27.0.1; IBM Corp., Armonk, NY, USA). Clinicopathological variables were summarized using absolute frequencies, percentages, means, and medians, as appropriate. Associations between clinicopathological variables and protein or transcription factor expression were evaluated using the χ^2^ test or Fisher’s exact test, when appropriate. Disease-specific survival (DSS) was defined as the interval between treatment completion and death attributable to OSCC. Patients who were alive at the last follow-up or who died from causes unrelated to OSCC were censored. Overall survival (OS) was defined as the interval from treatment to death from any cause or last follow-up. Survival curves were estimated using the Kaplan–Meier method and compared using the log-rank test. Hazard ratios (HRs) and 95% confidence intervals (CIs) were calculated using univariate and multivariate Cox proportional hazards regression models. Variables with *p* < 0.10 in univariate analyses were included in multivariate Cox regression models. All statistical tests were two-sided, and *p*-values < 0.05 were considered statistically significant.

## 5. Conclusions

Our findings support the relevance of EMT-related alterations in OSCC progression, particularly vimentin expression and complete EMT phenotypes, which were associated with aggressive clinicopathological features and poorer survival. However, the prognostic value of EMT-related biomarkers appears to be strongly influenced by established clinicopathological variables, especially clinical stage. Further validation in larger prospective cohorts integrating molecular approaches is warranted.

## Figures and Tables

**Table 1:** Expression of E-cadherin, N-cadherin, and vimentin and their associations with clinicopathological variables in a cohort of 165 OSCC patients.

Variable	Number (%)	E-Cadherin (%)		*p*	N-Cadherin (%)		*p*	Vimentin (%)		*p*
		(−)	(+)		(−)	(+)		(−)	(+)	
Gender										
Men	113 (69)	48 (43)	64 (57)	0.27	108 (97)	3 (3)	0.63	67 (60)	45 (40)	0.83
Women	52 (31)	27 (52)	25 (48)		49 (98)	1 (2)		32 (61)	20 (39)	
Tobacco use										
Smoker	107 (65)	45 (42)	61 (58)	0.25	103 (98)	2 (2)	0.61	67 (63)	39 (37)	0.31
Non-smoker	58 (35)	30 (52)	28 (48)		54 (96)	2 (4)		32 (55)	26 (45)	
Alcohol use										
Drinker	89 (54)	35 (40)	53 (60)	0.09	73 (99)	1 (1)	0.62	55 (63)	33 (37)	0.54
Non-drinker	76 (46)	40 (53)	36 (47)		84 (97)	3 (3)		44 (58)	32 (42)	
pT										
pT1 + 2	114 (73)	48 (42)	65 (58)	0.65	110 (99)	1 (1)	0.06	73 (64)	41 (36)	0.29
pT3 + 4	43 (27)	20 (46)	23 (54)		39 (93)	3 (7)		23 (55)	19 (45)	
pN										
pN0	95 (60)	39 (41)	56 (59)	0.27	91 (97)	3 (3)	0.48	66 (69)	29 (31)	0.002
pN+	63 (40)	31 (50)	31 (50)		60 (98)	1 (2)		28 (45)	34 (55)	
Clinical stage										
I + II	88 (53)	34 (39)	54 (61)	0.03	85 (99)	1 (1)	0.33	61 (69)	27 (31)	0.009
III + IV	77 (47)	41 (54)	35 (46)		72 (96)	3 (4)		38 (50)	38 (50)	
G status										
Well	105 (64)	46 (44)	58 (56)	0.61	102 (100)	0 (0)	0.01	73 (70)	32 (30)	0.001
Moderate-poor	60 (36)	29 (48)	31 (51)		55 (93)	4 (7)		26 (44)	33 (56)	
Tumor recurrence										
No	83 (50)	33 (40)	49 (60)	0.15	79 (99)	1 (1)	0.62	61 (73)	22 (27)	0.001
Yes	82 (50)	42 (51)	40 (49)		78 (96)	3 (4)		38 (47)	43 (53)	
Clinical status at the end of the follow-up										
Alive	98 (59)	41 (42)	56 (58)	0.28	94 (99)	1 (1)	0.30	69 (70)	29 (30)	0.001
Dead of index cancer	67 (41)	34 (51)	35 (49)		63 (96)	3 (4)		30 (45)	36 (55)	

**Table 2:** Expression of Snail1, Slug, ZEB1, Twist, and E47 and their associations with clinicopathological variables in a cohort of 165 OSCC patients.

Variable	Snail1 (%)		*p*	Slug (%)		*p*	ZEB1 (%)		*p*	Twist (%)		*p*	E47 (%)		*p*
	(−)	(+)		(−)	(+)		(−)	(+)		(−)	(+)		(−)	(+)	
Gender															
Men	39 (35)	74 (65)	0.02	16 (14)	97 (86)	0.08	111 (98)	2 (2)	0.18	6 (5)	106 (95)	1.0	109 (96)	4 (4)	0.67
Women	28 (54)	24 (46)		13 (25)	39 (75)		49 (94)	3 (6)		3 (6)	49 (94)		49 (94)	3 (6)	
Tobacco use															
Smoker	36 (34)	71 (66)	0.01	14 (13)	93 (87)	0.04	105 (98)	2 (2)	0.34	4 (4)	102 (96)	0.28	104 (97)	3 (3)	0.24
Non-smoker	31 (53)	27 (47)		15 (26)	43 (74)		55 (95)	3 (5)		5 (9)	53 (91)		54 (93)	4 (7)	
Alcohol use															
Drinker	32 (36)	57 (64)	0.18	10 (11)	79 (89)	0.02	88 (99)	1 (1)	0.18	2 (2)	86 (98)	0.08	85 (95)	4 (5)	1.0
Non-drinker	35 (46)	41 (54)		19 (25)	57 (75)		72 (95)	4 (5)		7 (9)	69 (91)		73 (96)	3 (4)	
pT															
pT1 + 2	42 (37)	72 (63)	0.26	12 (11)	102 (89)	0.001	112 (98)	2 (2)	0.12	4 (4)	109 (96)	0.11	111 (97)	3 (3)	0.09
pT3 + 4	20 (47)	23 (53)		14 (33)	29 (67)		40 (93)	3 (7)		5 (12)	38 (88)		39 (91)	4 (9)	
pN															
pN0	39 (41)	56 (59)	0.56	13 (14)	82 (86)	0.36	92 (97)	3 (3)	0.66	3 (3)	91 (97)	0.44	91 (96)	4 (4)	1.0
pN+	23 (37)	40 (63)		12 (19)	51 (81)		61 (97)	2 (3)		4 (6)	59 (94)		60 (95)	3 (5)	
Clinical stage															
I + II	32 (36)	56 (64)	0.23	8 (9)	80 (91)	0.002	86 (98)	2 (2)	0.66	2 (2)	85 (98)	0.05	85 (97)	3 (3)	0.70
III + IV	35 (46)	42 (54)		21 (27)	56 (73)		74 (96)	3 (4)		7 (9)	70 (90)		73 (95)	4 (5)	
G status															
Well	44 (42)	61 (58)	0.65	18 (17)	87 (83)	0.84	102 (97)	3 (3)	1.0	5 (5)	99 (95)	0.72	101 (96)	4 (4)	0.70
Moderate–poor	23 (38)	37 (62)		11 (18)	49 (82)		58 (97)	2 (3)		4 (7)	56 (93)		57 (95)	3 (5)	
Tumor recurrence															
No	29 (35)	54 (65)	0.13	11 (13)	72 (87)	0.14	81 (98)	2 (2)	0.68	3 (4)	79 (96)	0.49	81 (98)	2 (2)	0.27
Yes	38 (46)	44 (54)		18 (22)	64 (78)		79 (96)	3 (4)		6 (7)	76 (93)		77 (94)	5 (6)	
Clinical outcome															
Alive	38 (39)	60 (61)	0.56	14 (14)	84 (86)	0.17	95 (97)	3 (3)	1.0	4 (4)	93 (96)	0.48	95 (97)	3 (3)	0.44
Dead of index cancer	29 (43)	38 (57)		15 (22)	52 (78)		65 (97)	2 (3)		5 (7)	62 (93)		63 (94)	4 (6)	

## Data Availability

All data generated or analyzed in this study are provided within the article. Additional information is available from the corresponding author upon request.

## Abbreviations

The following abbreviations are used in this manuscript:

OSCC	Oral squamous cell carcinoma
EMT	Epithelial–mesenchymal transition
EMT-TFs	Transcription factors capable of inducing EMT
DSS	Disease-specific survival
OS	Overall survival
HR	Hazard ratio
CI	Confidence interval
